# End-diastolic forward flow (EDFF) after tetralogy of Fallot repair: Evolution of its role in management and prognosis

**DOI:** 10.1016/j.ijcchd.2026.100667

**Published:** 2026-03-10

**Authors:** Petru Baneu, Arie P. van Dijk, Anthonie L. Duijnhouwer, Tim ten Cate, Willem A. Helbing, Robin Nijveldt, Jo M. Zelis

**Affiliations:** a Department of Cardiology, Radboud University Medical Center, Geert Grooteplein Zuid 10, Nijmegen, 6525, GA, the Netherlands; b Department of Pediatric Cardiology, Erasmus Medical Center, Dr. Molewaterplein 40, 3015, GD, Rotterdam, the Netherlands

**Keywords:** Tetralogy of Fallot, Right ventricular restrictive physiology, End-diastolic forward flow, Prognostic factor, Multifactorial hemodynamic marker

## Abstract

**Objective:**

In the era of personalized medicine and multimodality imaging, renewed attention is directed toward the restrictive right ventricular (RV) physiology, including its historic hallmark, the end-diastolic forward flow (EDFF), in Tetralogy of Fallot (ToF) repair. Initially perceived as a marker of favorable restriction, EDFF has been repositioned as a multifactorial hemodynamic phenomenon rather than a specific indicator of myocardial stiffness, based on recent imaging and mechanistic studies.

**Methods:**

This review synthesizes the evolution of perception, the diagnostic assessment, and the prognostic relevance of EDFF in repaired ToF. A comprehensive PubMed search spanning from 1987 to 2024 identified relevant studies, including clinical trials, observational analyses, and meta-analyses on EDFF and RV restrictive physiology.

**Results:**

EDFF was initially associated with smaller “protective” RVs and improved exercise tolerance in early echocardiographic studies. Later, a dual-restrictive model distinguishing beneficial (primary) from maladaptive (secondary) patterns was proposed, with the addition of cardiac magnetic resonance (CMR). EDFF has recently been demonstrated to also occur in non-restrictive RV due to altered RV-pulmonary arterial coupling, regurgitant flow dynamics, or atrioventricular dyssynchrony. Novel 4D-flow imaging has enriched the general understanding of RV restrictive physiology but has not added significant prognostic value to EDFF.

**Conclusions:**

Advances in imaging and physiology have shifted EDFF from a unidimensional marker of restriction to a complex manifestation of right atrio-ventriculo-arterial interaction. While mechanistically informative, EDFF appears to lack prognostic specificity in isolation. Future research aimed at establishing standardized definitions that integrate volumetric, kinetic, and coupling parameters could enhance the diagnostic and clinical relevance.

## Abbreviations

2D-flow: Two-Dimensional Phase-Contrast Flow Imaging
4D-flow: Four- Dimensional Phase-Contrast Flow Imaging
AHA: American Heart Association
CMR: Cardiac Magnetic Resonance
EDFF: End-Diastolic Forward Flow
ESC: European Society of Cardiology
ICU: Intensive Care Unit
LGE: Late Gadolinium Enhancement
LV: Left Ventricle
PR: Pulmonary Regurgitation
PV: Pulmonary Valve
RV: Right Ventricle
RVEDP: Right Ventricular End-Diastolic Pressure
RVOT: Right Ventricular Outflow Tract
SD: Standard Deviation
TAP: Transannular Patch
ToF: Tetralogy of Fallot
VTI: Velocity-Time Integral

## 1 Introduction

Tetralogy of Fallot (ToF), first described by Étienne-Louis Arthur Fallot in 1888, is the most common cyanotic congenital heart disease beyond the neonatal period and a key milestone in the evolution of congenital cardiac surgery [1,2]. Surgical advances, including Blalock's systemic-to-pulmonary shunt and Lillehei's intracardiac repair, enabled long-term survival and the emergence of large adult cohorts, shifting clinical focus toward long-term outcomaes and management [3]. As right ventricular (RV) systolic function often remains preserved, diastolic function has emerged as a critical determinant of adaptation and prognosis [4]. Interpretation of RV diastolic behavior is further complicated by its dual embryologic origin, conferring distinct hybrid pump–conduit properties [5,6].

Early catheterization studies identified elevated right ventricular end-diastolic pressures (RVEDP) and dip-and-plateau tracings, consistent with restrictive physiology [7,8]. Nonetheless, with the widespread adoption of Doppler echocardiography, end-diastolic forward flow (EDFF) in the pulmonary artery became the principal non-invasive marker linking invasive hemodynamics to clinical assessment [9,10].

This review examines the evolving concept of EDFF in repaired tetralogy of Fallot, highlighting its shift from a marker of favorable restriction toward a more complex, multifactorial phenomenon and discussing its implications for prognosis and management.

## 2 Methods

A comprehensive literature search was conducted in the PubMed database using the terms “end-diastolic forward flow”, “late diastolic antegrade flow”, “restrictive right ventricular physiology”, “tetralogy of Fallot,” and “4D-flow”. A total of 67 publications from 1987 to 2024 were identified, including original research articles, randomized clinical trials, observational studies, systematic reviews, and meta-analyses. Among these, studies providing significant historical data or novel insights were selected for inclusion in this narrative review. EDFF was initially referred to as late diastolic antegrade flow, but in the early 2010s, the terminology shifted to the more precise “end-diastolic forward flow”. For clarity and consistency, we will adopt this accepted terminology throughout the review.

## 3 Early perceptions (1990s): EDFF as a hallmark of protective restriction with favorable mid-term prognosis

**Fig. 1:**
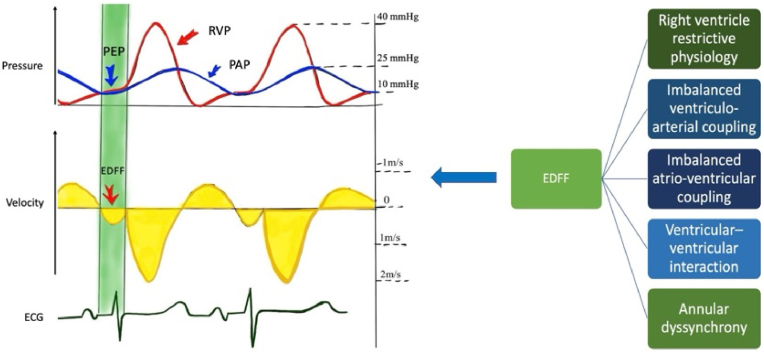
Schematic representation of right heart catheterization tracings correlated with trans-pulmonary Doppler echocardiography and ECG, illustrating the atrial systole responsible for generating the **EDFF** at the moment of pressure equalization between the right ventricle and the pulmonary artery; **EDFF**: End-diastolic forward flow, **PEP**: Pressure equalization point, **PAP**: Pulmonary arterial pressure, **RVP**: Right ventricular pressure.

The first description of this phenomenon dates to 1987, when Kisanuki et al. investigated patients after pulmonary valvotomy for isolated pulmonary stenosis using Doppler echocardiography, corroborated by right heart catheterization [10]. They identified a late diastolic antegrade flow across the pulmonary valve in three patients, occurring immediately after the P wave and corresponding to a dip-and-plateau RV pressure tracing [10]. This was interpreted as an atrial systole transiently increasing RV pressure above pulmonary artery diastolic pressure in a noncompliant RV, thereby briefly opening the pulmonary valve and generating antegrade flow, as illustrated in Fig. 1 [10].

In 1988, Appleton et al. characterized restrictive ventricular physiology through transmitral and transtricuspid Doppler analysis, describing shortened E-wave deceleration times, tall A-waves, and reduced early diastolic filling in patients with clinical and hemodynamic evidence of restriction [11]. Their analysis, however, was limited to atrioventricular inflow and did not assess pulmonary valve flow; consequently, EDFF was not identified [11].

Redington et al. subsequently integrated these concepts in 1992 by studying patients after repair of pulmonary atresia with an intact ventricular septum [12]. Using Doppler interrogation of the atrioventricular and semilunar valves, they demonstrated pulmonary artery EDFF in 6 of 7 patients in sinus rhythm but in none of the controls [12]. Although all EDFF - positive patients showed apparently normal tricuspid E/A patterns, a universally present pulmonary regurgitation (PR), but without reported severity, limits the interpretation of the relationship between EDFF and transtricuspid filling in this cohort [12].

Together, these early studies established RV diastolic function as comprising two phases, as suggested by earlier invasive observations: an early phase reflected by transtricuspid inflow patterns, and a late phase characterized by EDFF across the pulmonary valve as a marker of restriction [[10], [11], [12]]. In the studies by Kisanuki and Redington, EDFF was consistently associated with restrictive physiology, either confirmed invasively or inferred in surgically treated pulmonary atresia with a small, fibrotic RV [11,12]. Consequently, EDFF became regarded as a reliable non-invasive marker of RV diastolic dysfunction, largely independent of early filling indices.

In 1995, Cullen et al. evaluated the clinical impact of EDFF in 35 patients after ToF repair and demonstrated an association with adverse early postoperative outcomes, including prolonged ICU stay, higher inotropic requirements, and increased complications [13]. Paradoxically, EDFF was also associated with favorable hemodynamic effects, such as increased stroke volume and shorter PR duration, leading the authors to propose ventilatory strategies aimed at preserving this compensatory mechanism [13]. In the same year, Gatzoulis et al. examined long-term ToF survivors 15–35 years after repair and reported superior exercise capacity and smaller cardiothoracic ratios in patients with restrictive RV physiology [14]. They proposed that RV restriction may be protective by limiting PR, augmenting stroke volume, and preventing progressive RV dilation, without adversely affecting right atrial pressures due to concomitant retrograde superior vena cava flow [14].

While studies by Kisanuki and Appleton proposed EDFF as a marker of restrictive RV physiology, Cullen and Gatzoulis took it a step further. Instead of just exploring this link, they made EDFF a central element in the pathophysiology of repaired ToF, associating it with a favorable prognosis [10,11,13,14].

It is worth noting that in children, a small antegrade pulmonary flow during inspiration can be a normal physiological finding, indicating enhanced venous return and atrial contraction. [13,14]. To prevent misclassification, Gatzoulis et al. defined EDFF as present throughout the respiratory cycle to be considered pathological [[13], [14], [15], [16], [17]].

Other studies from 1996 to 1999, reinforced the idea of restrictive RV physiology as a protective remodeling pattern with a favorable mid-term prognosis, identified by the presence of EDFF. In addition to the known benefits, it was also linked to less QRS prolongation and a lower arrhythmic burden and was found to occur more often in patients repaired with a transannular patch (TAP) [15,16].

To sum up, between 1995 and 1999, the foundations of the EDFF concept were established and widely accepted in the literature as a hallmark of restrictive physiology and protective remodeling pattern with a favorable mid-term prognosis [14,17]. The term "mid-term” is important to understand, as it refers to a median follow-up of 7.4 years across these studies, or 10.5 years when including the broader 15–35-year range reported by Gatzoulis et al. Restriction was consistently linked to smaller RV size, shorter PR, less QRS prolongation, and fewer arrhythmias, creating the impression of a favorable remodeling phenotype [14,17].

## 4 Transitional period (late 1990s – early 2010s): Conflicting evidence, emerging complexities, development of the dual-phenotype restrictive model

While one side of the scientific community was establishing EDFF as a hallmark of favorable restrictive RV physiology, new studies began to present conflicting findings. A key study was by Helbing et al., in 1997, which for the first time incorporated cardiovascular magnetic resonance (CMR) into the evaluation of EDFF and restrictive physiology [18]. Their research analyzed both transtricuspid and transpulmonic flow patterns, quantified RV volumes, and correlated these with transvalvular Doppler measurements. In patients with smaller RVs, they confirmed earlier findings of EDFF; however, in those with dilated ventricles, they identified impaired early filling patterns. To put this in context: since Appleton in 1988, a shortened E-wave deceleration time with tall A-waves has been considered the classic Doppler marker of restriction [11]. Yet, Redington later showed normal tricuspid inflow in patients with EDFF, and subsequent ToF studies either ignored tricuspid inflow or did not incorporate it, almost exclusively associating diastolic dysfunction with small, restrictive RVs and EDFF [12,[14], [15], [16], [17]]. By adding RV volumetric analysis and revisiting tricuspid inflow, Helbing's work demonstrated that a restrictive pattern can also appear - albeit at the level of early diastolic filling - in dilated RVs, thus challenging the traditional one-dimensional view of restriction in ToF [[14], [15], [16], [17], [18]].

In 2006, Vukomanović et al. proposed a dual model of restrictive physiology that effectively combined the findings of all previous studies. The first subtype, called “primary restrictive physiology,” described a small, non-compliant RV characterized by EDFF, consistent with Gatzoulis protective model. The second subtype, “secondary restrictive physiology,” referred to a dilated RV affected by chronic volume overload, mainly caused by severe PR and a restrictive transtricuspid inflow pattern - an idea previously suggested by Helbing's observations [14,18,19]. This model was later confirmed by studies in subsequent years that incorporated CMR volumetry, especially those by Lee and Samyn in 2013 [[19], [20], [21]]. Both studies noted that patients with relatively small RV volumes (<170 mL/m^2^) and EDFF - the “primary” restrictive pattern - showed higher exercise capacity, indicating a protective effect. Conversely, patients with significantly dilated RVs and EDFF no longer gained functional benefits, aligning with the “secondary” restrictive phenotype. Importantly, while Lee explicitly linked these patterns to prognosis through exercise capacity and functional performance, Samyn demonstrated that in younger, mostly asymptomatic groups repaired with modern surgical techniques, restriction was mainly related to PR and RV dilation, with early diastolic strain abnormalities potentially signaling future maladaptation [21,22]. Together, these findings support Vukomanović’s dual framework and highlight its prognostic value across different patient populations [21,22].

To conclude and integrate all the findings so far, the different interpretations and seemingly conflicting evidence regarding the triad EDFF - restrictive RV physiology - prognosis in surgically repaired ToF patients can be summarized as follows. In the 1960s and 1970s, extensive ventriculotomies, large TAPs, long ischemia times, and limited myocardial protection resulted in heavily scarred and fibrotic right ventricles that became restrictive soon after repair [3]. In this context, with pulmonary valve function compromised and RVs almost always stiff, ventricular size played a key role. A smaller cavity increased the restrictive component and, aided by EDFF, boosted stroke volume and reduced PR [11,12]. These positive hemodynamic effects led to better exercise tolerance and mid-term outcomes. Conversely, when the ventricle was already dilated and stiff, even if EDFF was present, it no longer provided a significant protective effect [3,11,12]. In newer cohorts, repaired in the 1980s and 1990s with advanced techniques and better myocardial preservation, the RV was less affected by surgical scarring and initially remained more compliant, though it was chronically exposed to PR. In this case, initial size was less critical, since most ventricles were compliant and non-restrictive after repair; what mattered most was the severity of PR, which over time caused dilation, loss of compliance, restrictive physiology, and reduced exercise capacity, leading to worse prognosis [[22], [23], [24], [25]]. Therefore, in modern groups, smaller RVs were typically not restrictive, while dilated RVs eventually became restrictive, with restriction being linked to poor outcomes. In summary, it was not the restrictive physiology itself that determined prognosis but rather the context in which it occurred: early restriction in a smaller RV was associated with favorable hemodynamics and better outlook, while restriction later in a dilated RV indicated maladaptive remodeling and was linked to worse prognosis [3,[10], [11], [12], [13], [14], [15], [16], [17], [18],[22], [23], [24], [25]].

One valid question arises: why is the RV prone to become restrictive over time due to chronic volume overload from severe PR, while the LV, facing a similar burden in severe aortic regurgitation, tends to stay dilated yet still compliant even with substantial regurgitant volumes? As Ganni et al. highlight, chronic RV volume overload occurs in a myocardial environment that cannot mount an adequate angiogenic response: capillary density fails to expand proportionally to hypertrophy, while mitochondrial dysfunction and oxidative stress impair energy supply. This mismatch fosters hypoxia-like conditions, maladaptive extracellular matrix remodeling, and progressive diffuse myocardial fibrosis, reducing compliance [26]. Symakani et al. further point out a structural vulnerability: the RV's unique myoarchitecture, with its middle circumferential trabecular layer, responds differently to mechanical stress than the LV [27]. Subjected to sequential stressors typical of ToF (initial pressure overload, hypoxemia, and later chronic volume overload), this architecture predisposes the RV to maladaptive stiffening rather than adaptive dilation. It must be mentioned, however, that in a study published in 2021, where serial LGE-CMR assessments of the RVs were effectuated with a follow-up of 4 years in a ToF subpopulation, no significant progression of LGE pattern was described. This suggest that diffuse myocardial fibrosis rather than replacement fibrosis, might be a more plausible substrate for a dilated RV that evolves toward restrictive physiology at later stages [28]. Together, these structural, microvascular, and bioenergetic limitations explain why the RV shifts to a restrictive state under chronic regurgitant load, in sharp contrast to the LV's ability for sustained compliant dilation [26,27].

## 5 Contemporary perception (mid-2010s-present): evidence challenging the dual-phenotype restrictive model, EDFF – multifactorial etiology

If the dual-phenotype restrictive model suggested that the presence of EDFF could indicate both good and bad prognosis, depending on the type of restrictive physiology involved, four years later, Mori et al., in 2017 went even further by providing the first invasive evidence that EDFF is not always synonymous with restrictive physiology [29]. Using simultaneous pressure recordings in the right atrium, right ventricle, and pulmonary artery, they distinguished between true restrictive physiology and what they called a pseudo-restrictive pattern. Unlike the dual-phenotype restrictive model, Mori observed that some patients with significantly enlarged RVs showed EDFF despite normal right atrial pressures and no increase in RV end-diastolic pressure. In these cases, EDFF occurred alongside the equalization of RV and pulmonary artery diastolic pressures at end-diastole, leading to premature valve opening [29]. This hemodynamic profile was incompatible with a truly stiff RV and therefore indicated a pseudo-restrictive physiology, where EDFF reflects regurgitant flow paths rather than intrinsic myocardial restriction. Mori suggested that when PR is severe enough, even a compliant, dilated RV - evidenced by normal atrial pressures - may not be able to handle the large amount of regurgitant volume, resulting in a brief rise in RV end-diastolic pressure above pulmonary artery diastolic pressure and generating EDFF in a non-restrictive ventricle [29]. Symakani et al., in a recent study on a porcine model in which pulmonary stenosis was surgically induced and subsequently relieved with a TAP producing free PR, demonstrated that chronic severe PR leads to a marked increase in pulmonary arterial elastance. Although their study did not specifically assess EDFF, these findings support the broader concept that severe PR can profoundly modify RV–PA diastolic hemodynamics even in the absence of true restrictive physiology [30].

One year later, Kutty et al. expanded on this idea, further dissociating EDFF from restriction by demonstrating its presence without right atrial enlargement or elevated right atrial pressures [31]. Instead, EDFF was linked to higher RV stroke volume and reduced LV end-diastolic volume, indicating that ventricular–ventricular interaction and altered loading conditions, rather than impaired RV compliance, could be key factors in certain situations [30]. Moreover, several years earlier, Sun et al. had already shown that delayed tricuspid inflow and annular dyssynchrony could also cause EDFF independently of atrial pressures or myocardial stiffness, suggesting it could also be a timing-related phenomenon rather than only a strict marker of restriction [32].

Overall, in just five years (2013–2018), the dual-phenotype restrictive model proved insufficient, as increasing evidence indicated that interventricular interaction, imbalanced ventriculo-arterial coupling, and annular dyssynchrony could produce EDFF even in non-restrictive RVs [[29], [30], [31], [32]]. Therefore, EDFF should be seen not only as a marker of restrictive physiology but also as one of several potential signs of a broader right atrio–ventriculo–arterial hemodynamic imbalance.

## 6 Definitional ambiguities and inconsistencies of EDFF and restrictive RV physiology

Pijuan-Domènech et al., in 2021, evaluated by transthoracic echocardiography 81 adults with repaired RVOT lesions (64% ToF) and 43 controls, demonstrating for the first time that mild EDFF can occur in healthy subjects. Patients showed higher EDFF velocities, longer durations, and greater respiratory variability. Quantitative cut-offs (>2 SD above control means) defined pathological EDFF, characterized by respiratory independence. EDFF magnitude correlated with PR fraction on CMR, indicating it reflects PR severity and RV–PA coupling rather than intrinsic myocardial stiffness. This study provided the first systematic calibration of EDFF in healthy adults and clearly distinguished EDFF from restrictive RV physiology as nearly synonymous concepts [33].

Based on this perspective, an analysis of the most relevant papers discussing EDFF in the context of restrictive RV physiology over the past 30 years shows significant variation in its definition. Some authors described EDFF as a late antegrade “a-wave” detectable throughout the entire respiratory cycle, while others required its persistence over at least three, four, or even five consecutive cardiac cycles. Some groups focused on a velocity threshold for the late diastolic antegrade wave or measured EDFF as a minimum percentage of total forward flow. In certain studies, these criteria were combined, whereas others used a straightforward binary method where simply detecting EDFF was considered enough. Notably, when restrictive RV physiology was defined, it was almost always equated with the presence of EDFF - although based on the highly variable criteria described above [[14], [15], [16], [17], [18], [19], [20], [21], [22], [23], [24], [25], [26], [27], [28], [29], [30], [31], [32], [33], [34], [35], [36], [37], [38], [39], [40], [41]].

Therefore, as suggested in the 2022 meta-analysis by Van den Eynde et al., the current understanding can be summarized as follows: a non-invasive marker - detected by Doppler echocardiography or 2D-flow CMR - that is sometimes physiologically present in both children and adults, without a standardized definition, used with highly variable measurement methods, and with a demonstrated multifactorial cause (as illustrated in Fig. 1), has been equated with restrictive RV physiology for nearly thirty years. Notably, the original proposal by Gatzoulis in 1995 - that pathological EDFF should be persistent through the entire respiratory cycle - is applicable to Doppler echocardiography but hardly to CMR techniques, which require breath-holding for accurate 2D-flow measurement. This methodological limitation helps explain the complex and often conflicting prognostic associations reported in relation to restrictive RV physiology in repaired ToF patients during this period [[14], [15], [16], [17], [18], [19], [20], [21], [22], [23], [24], [25], [26], [27], [28], [29], [30], [31], [32], [33], [34], [35], [36], [37], [38], [39], [40], [41], [42]].

## 7 EDFF in the current context of novel imaging modalities

Novel imaging techniques such as 4D-flow CMR were quickly adopted for evaluating repaired ToF patients. Their main advantage lies in the ability to quantify flow dynamics and kinetic energy efficiency, providing mechanistic insights. In this context, Sjöberg et al., in 2018 first used 4D-flow to demonstrate that restrictive RVs (defined by EDFF) were smaller and more energy-efficient, yet this apparent hemodynamic benefit did not lead to better exercise tolerance, raising questions about its clinical importance [38]. More recently, Zhao et al., in 2024, emphasized the multifactorial nature of EDFF, showing that it can occur without restrictive physiology and should be seen as one of several expressions of complex atrio-ventriculo-arterial interactions, thus challenging its role as a specific prognostic marker [39]. Voges et al. also found that restrictive patients exhibited altered diastolic kinetic energy patterns, but again, these changes were not clearly associated with clinical outcomes [41]. Overall, these studies demonstrate that while 4D-flow has enhanced the mechanistic understanding of RV hemodynamics, it has not clarified the uncertainties surrounding EDFF, and its prognostic value remains inconsistent and debated [38,41,43].

What 4D-flow CMR has contributed, however, is shedding light on the initial 1995 Gatzoulis model of a relatively small restrictive RV by explaining its more efficient hemodynamics [14]. According to the Law of Laplace, a smaller cavity radius in a rigid ventricle lowers wall stress for any given intracavitary pressure. In this configuration, less energy is dissipated into chamber distension, and a greater proportion of atrial systolic impulse and diastolic recoil is transmitted into forward flow [44]. This provides a structural explanation for the paradoxical efficiency of restrictive physiology in small RVs, with less wasted kinetic energy in regurgitant jets. Sjöberg et al., using 4D-flow CMR, confirmed this principle by demonstrating that this type of RV was energetically more efficient, with lower diastolic kinetic energy despite similar PR severity [38,44]. Thus, their work offered direct mechanistic validation of the hemodynamic efficiency implied by Gatzoulis’ restrictive model [14,38,44].

## 8 EDFFs current prognostic value

The goal of disease prognostication is to enable proper, timely, and effective intervention during its course. For the repaired ToF subpopulation, this mainly involves managing residual PR and timing valve replacement appropriately [1,5]. EDFF's limited reproducibility and prognostic specificity are reflected in its absence as a factor in PVR decision-making in the latest ACC/AHA and ESC congenital heart disease guidelines [1,2]. Additionally, Mayourian et al., in their mortality risk score for repaired ToF derived from the INDICATOR cohort, also excluded EDFF [45,46]. Conversely, factors such as indexed ventricular volumetry, type of surgery, biventricular function, markers of atrial loading, electrical remodeling, arrhythmia burden, and fibrosis burden as indicated by LGE-CMR were considered essential or added prognostic value [45,46].

## 9 Future perspectives

Although EDFF is no longer a variable in the prognostic model for this specific patient subgroup, it still undeniably functions as a mechanistic marker with potential value when properly calibrated and interpreted within the appropriate context. Additionally, restrictive RV physiology continues to be both a clinical reality and a useful concept. Moving forward, efforts should aim to develop a more precise and standardized model of RV restriction that considers the complex atrioventricular, ventriculo-arterial, and interventricular interactions, while also incorporating kinetic energy profiles measured by 4D-flow. Such a strategy could improve reproducibility and offer meaningful additional prognostic insights.

## 10 Conclusion

This review highlights not only the evolving understanding of restrictive RV physiology and EDFF in repaired tetralogy of Fallot, but also a broader phenomenon in medical science: discoveries, once made, are often taken for granted and carried forward into new eras without re-examining their validity under changing paradigms. By tracing how EDFF shifted from being viewed as a protective hallmark to a marker of heterogeneous phenotypes and a pseudo-restrictive epiphenomenon in some contexts, we emphasize the necessity of continuously re-validating concepts as diagnostic tools, imaging modalities, and patient populations evolve.

## CRediT authorship contribution statement

**Petru Baneu:** Conceptualization, Investigation, Writing – original draft, Writing – review & editing. **Arie P. van Dijk:** Data curation, Methodology, Supervision. **Anthonie L. Duijnhouwer:** Data curation, Methodology, Visualization. **Tim ten Cate:** Data curation, Formal analysis, Supervision. **Willem A. Helbing:** Supervision, Validation. **Robin Nijveldt:** Data curation, Formal analysis, Supervision, Validation. **Jo M. Zelis:** Conceptualization, Investigation, Methodology.

## Ethical approval

Not applicable.

## Funding statement

This research received no specific grant from any funding agency in the public, commercial, or not-for-profit sectors.

## Declaration of competing interest

The authors declare that they have no known competing financial interests or personal relationships that could have appeared to influence the work reported in this paper.
